# Mechanisms of Transsynaptic Degeneration in the Aging Brain

**DOI:** 10.14336/AD.2024.03019

**Published:** 2024-10-01

**Authors:** Roshana Vander Wall, Devaraj Basavarajappa, Alexander Klistoner, Stuart Graham, Yuyi You

**Affiliations:** ^1^Faculty of Medicine Health and Human Sciences, Macquarie University, North Ryde, NSW, 2109, Australia.; ^2^Save Sight Institute, Sydney University, Sydney, NSW, 2000, Australia.

**Keywords:** Transsynaptic Degeneration, Glia, Senescence, Neurodegenerative Disease, Aging

## Abstract

A prominent feature in many neurodegenerative diseases involves the spread of the pathology from the initial site of damage to anatomically and functionally connected regions of the central nervous system (CNS), referred to as transsynaptic degeneration (TSD). This review covers the possible mechanisms of both retrograde and anterograde TSD in various age-related neurodegenerative diseases, including synaptically and glial mediated changes contributing to TDS and their potential as therapeutic targets. This phenomenon is well documented in clinical and experimental studies spanning various neurodegenerative diseases and their respective models, with a significant emphasis on the visual pathway, to be explored herein. With the increase in the aging population and subsequent rise in age-related neurodegenerative diseases, it is crucial to understand the underlying mechanisms of

## 1. Introduction

Neurodegenerative disorders are becoming increasingly prevalent with the growing aging population, a factor compounded by increased life-expectancy. The United Nations 2020 report on world population aging revealed that there were 727 million people aged 65 years or over worldwide. This demographic is anticipated to increase from 9.3 per cent in 2020 to around 16.0 per cent in 2050, surpassing 1.5 billion (https://digitallibrary.un.org/record/3898412/files/undesa_pd-2020_world_population_ageing_highlights.pdf). These statistics are put into harsh perspective when presented with the recent report that the age range with the highest incidence of neurodegenerative diseases is between 60-78 years [1].

Age poses a major risk factor for the development of neurodegenerative diseases, with progressive neuronal loss leading to either physical disability and/or cognitive deficits. Perceived concomitance makes it almost easy to accept that these diseases are a common symptom of aging, particularly in very elderly individuals. In addition to age being a developmental risk factor, it enhances disease severity and can hamper recovery from an episode [2, 3]. It is important to recognise the changes that occur in the aging brain and retina include neuroinflammation, cellular senescence, altered intercellular communication, reduced volume, leaky blood brain barriers (BBB), neurotransmission disruption, immune dysfunction, making it more susceptible to neurodegenerative diseases [4-7]. Many of these pathological changes can be seen in the more prevalent age-related neurodegenerative diseases such as multiple sclerosis (MS), Alzheimer’s disease (AD), Parkinson’s disease (PD), amyotrophic lateral sclerosis (ALS), glaucoma, optic neuritis and stroke [8-13]. A common pathology that arises across these conditions is the spread of damage from the initial injury site to new, anatomically and functionally connected regions in the CNS [14]. This phenomenon is known as transsynaptic degeneration (TSD) and is often observed as a prominent feature in neurodegenerative disease, one that exacerbates the disease and leads to clinical degradation [15, 16].

Due to the neuronal connectivity implications of degeneration, the use of a well-defined neurological pathway is essential to study TSD phenomenon. For this reason, the visual pathway is the most frequently used model, resulting in a long history of both retrograde and anterograde TSD documented in the visual pathway beginning as early as 1913 [17, 18]. The visual pathway’s unique, yet uncomplicated hierarchical neuronal architecture provides an ideal structure for studying this phenomenon, because of its more linear nature compared to other more multifarious neuronal pathways [19]. An additional benefit of utilising this pathway is that it is frequently affected in several neurodegenerative conditions, providing an opportunity to investigate and enhance our understanding of the progression of these diseases [20, 21].

Considering the limited understanding of TSD and its significant impact on neurodegenerative diseases, unravelling its mechanisms could lead to the development of a host of potential treatment strategies. This review will cover the mechanisms of TSD, specifically in the context of age-related neurodegenerative diseases, with a particular focus on glial and synaptically mediated degeneration.

## 2. Transsynaptic Degeneration in Neural Networks

Evidence for TSD was first described by Nissl, wherein he observed chromatolysis in anterior horn cells 24 h post peripheral nerve transection [22]. Subsequent studies went on to demonstrate this phenomenon, primarily utilising the visual pathway of non-human animals [17, 23, 24]. Following optic nerve injury, retinal ganglion cells (RGCs) displayed similar chromatolysis [25]. Retinal cells are the first portion of the visual system, with RGCs comprising the optic nerve which then synapses with second order neurons in the lateral geniculate nucleus (LGN), which in turn project the optic radiations (OR) to the visual cortex (V1) in the brain [26].

**Figure 1.**
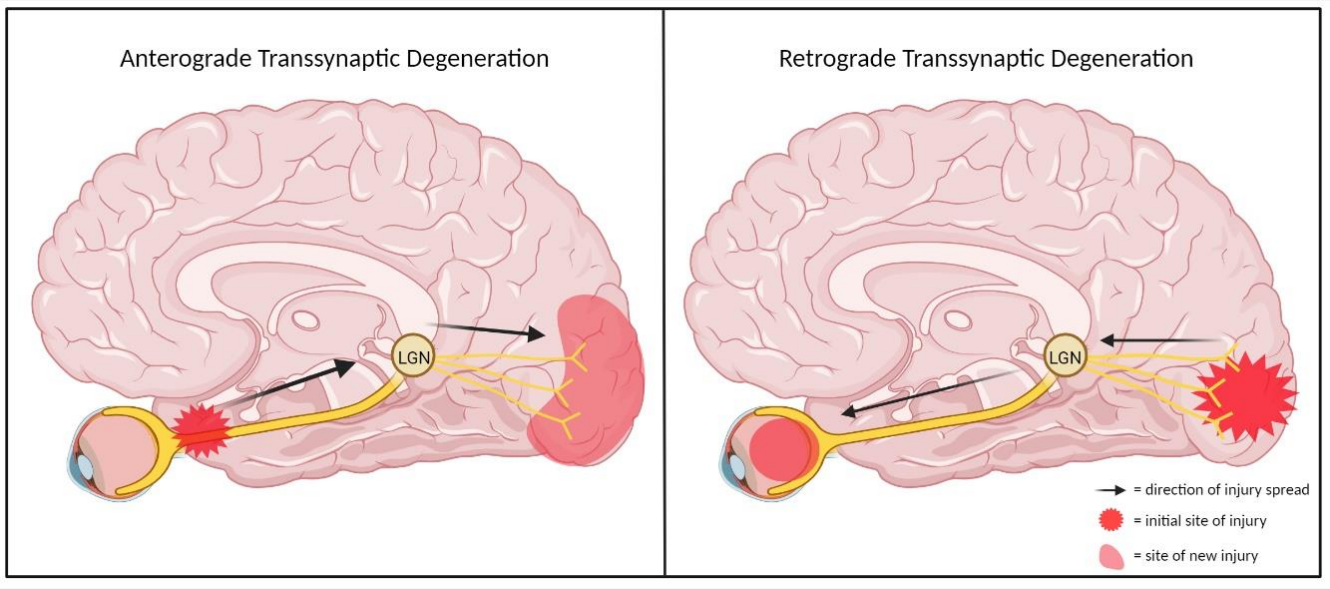
Transsynaptic degeneration in the visual pathway. TSD in the visual pathway can operate in anterograde or retrograde directions. Left panel: anterograde TSD - damage at the optic nerve spreading transsynaptically to the visual cortex. Right panel: retrograde TSD - damage at the visual cortex spreading transsynaptically to the retina.

Degeneration of neurons can occur in either an anterograde or retrograde fashion following injury to cells with which they form synapses. For instance, RGC damage in glaucoma has been demonstrated to cause injury anterogradely to the LGN and even the V1. Conversely, retinal degeneration can be seen after sustaining damage to the V1 region of the brain (Fig. 1) [9].

### 2.1. The Visual Pathway Exhibits Retrograde and Anterograde TSD

One of the primary benefits of employing the visual pathway to study TSD and age-related disease progression is that both retro and anterograde degenerative changes can be observed using non-invasive techniques such as optical coherence tomography (OCT) and visual evoked potentials (VEPs) [27, 28]. These techniques are often combined with magnetic resonance imaging (MRI), though further refinement is required amongst all three to reliably track changes when exploring TSD [29].

A study conducted by Balk and colleagues utilising the visual pathway outlined the relationship between MS and bidirectional TSD [30]. This study showed a negative relationship between retinal nerve fibre layer (RNFL) thickness and lesions within the ORs. MS patients with optic neuritis (ON) commonly exhibit thinning of the RNFL, but more interestingly, MS patients without ON also develop RNFL thinning. This phenomenon is believed to be a result of retrograde TSD. Similarly, the converse anterograde TSD was also observed, where damage to the retina or optic nerve resulted in degradation in the ORs or LGN region of the brain, [10, 31]. This demonstrates that TSD process can move in either direction in the one pathology. One of the major contributors to axonal survival in both forms of TSD is glial-mediated support. However, defective glial activity or clearance of damaged axons can hinder axonal regeneration. This situation in turn provokes inflammation, a process thought to precede axonal loss [12, 32]. Despite similarities, it is unclear whether anterograde and retrograde TSDs share similar underlying mechanisms.

Early studies conducted by Ghetti and Wiśniewski demonstrated that after damage to the pre-synaptic LGN neurons, both pre-synaptic axon terminals and post-synaptic dendrites were engulfed by glial cells, presenting an additional mechanism for anterograde degeneration; Wallerian degeneration [33, 34]. This occurs when the soma of the neuron is damaged and this damage spreads to the axon as a lack of trophic support, leading to atrophy and eventual neuronal death [35, 36]. Numerous TSD studies focus on the visual pathway, with many studies discussed in this review centred on this aspect. However, some studies have explored TSD in other pathways affected by neurodegenerative diseases will also be discussed for broader relevance.

### 2.2. TSD in Other Commonly Affected Pathways

While the visual pathway serves as a prime model for TSD, many other neuronal networks are also affected [37]. One such instance is the anatomical neighbour of the visual pathway, the olfactory pathway. Anterograde TSD of the olfactory pathway is observed after primary insult to the first order neurons in the olfactory epithelium, resulting in morphologic changes to the olfactory bulb and tract [38]. Similarly to the visual pathway, it TSD can also manifest retrogradely as well, with insults to the orbital frontal cortex, parahippocampal gyrus and/or piriform cortex affecting the olfactory blub [39]. Another system that is affected by TSD is the corpus callosum. Connecting the hemispheres and with connections that reach from the parietal, temporal and occipital lobes, damage to the primary cortex can trigger TSD in the corpus callosum [40]. Even the limbic system is not immune to TSD, with damage to certain structures impacting the Papez circuit [41]. Formed by the hippocampus, fornix, mammillary body (MB), mammillary-thalamic tract, anterior thalamic nucleus, anterior thalamic radiation, cingulum, and parahippocampal gyrus, damage to the anterior thalamic nucleus results in retrograde TSD of the MB via the mammillary-thalamic tract, with anterograde TSD of the MB and fornix when injury is sustained by the hippocampus [42, 43]. There are hindbrain systems that are also impacted, though far less commonly. The corticopontocerebellar pathway and the dentate-rubro-olivary pathway both display signs of TSD when a region of their respective pathways is injured [44, 45].

In addition to CNS pathways that are affected by aforementioned neurodegenerative diseases, TSD also impacts various spinal pathways and subsequently the peripheral nervous system (PNS). Often utilised in models of PD, it has been shown that a number of spinal tracts subject to experimental TSD exhibit retrograde damage to their corresponding cortical regions [46, 47]. The corticospinal tract for instance, is the major neuronal pathway providing voluntary motor function. Injury to any portion of the corticospinal tract or the primary motor cortex can lead to Wallerian degeneration. In the case of TDP-43 ALS model, TSD progressed in an anterograde manner from the prefrontal cortex [13, 48]. There are numerous pathways within the CNS that are subject to TSD. TSD in the nigrostriatal pathway is often observed in PD patients, with degeneration of the dopaminergic neurons of the substantia nigra after injury of the striatum [11, 49].

Naturally, these pathways aren’t the only ones affected by TSD, which also makes it all the more devastating as it has the potential to affect any neuronal pathway. The varied mechanisms of TSD manifest in many age-related neurodegenerative diseases, and while not all occurrences of TSD are age-related, what is occurring in the aging brain could certainly contribute to disease development and progression.

## 3. Mechanisms of Transsynaptic Degeneration and Age-related Neurodegenerative Diseases

### 3.1. Changes in Neurons and Across the Synapse

#### 3.1.1. Neurotransmission and Excitotoxicity

**Figure 2.**
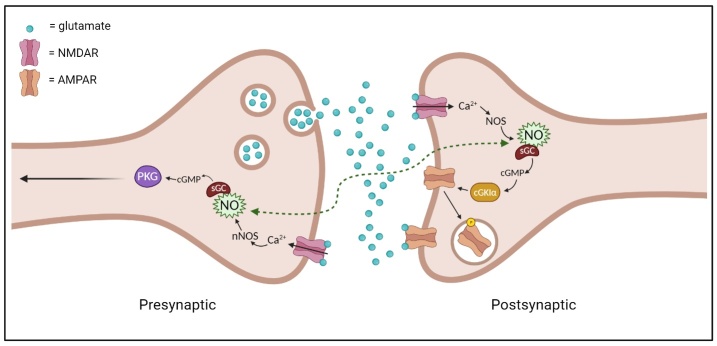
Postsynaptic nitric oxide (NO) mediated signalling. Endogenous NO synthesis, facilitated by soluble guanylyl cyclase (sGC) initiates cyclic guanosine monophosphate (cGMP) signalling in postsynaptic neurons. This stimulates endocytosis of glutamate receptor subtype, AMPA, diminishing synaptic transmission. Excess extracellular glutamate is taken back up by presynaptic NMDARs. In presynaptic neurons this activity produces the damaging molecule, protein kinase G (PKG).

Often taken for granted, neurotransmission is essential for normal neurological functioning. However, when it becomes dysregulated or halted, the knock-on effects can have a significant impact. During the synaptic transmission process, postsynaptic neuronal signalling plays a major role in neuronal communication. Fitzsimonds and Poo illustrated that back propagation of signalling across the synapse to the presynaptic neuron can influence transmitter release from presynaptic terminals, possibly preventing further activity [50, 51]. Therefore, similarly to anterograde TSD, a lack of signalling from postsynaptic neurons is a possible mechanism for retrograde TSD [52]. Alternatively, postsynaptic neurons can send potentially damaging signals or molecules towards the presynaptic terminal. In a back-propagation mechanism in AD conditions, Aβ1-42 inhibited the dendritic A-type K+ current in hippocampal CA1 pyramidal neurons [53]. This inhibitory back-propagation increased dendritic action potential amplitude via influx of Ca^2+^ resulting in destabilisation of Ca^2+^ homeostasis and subsequent neuronal death [54, 55]. Another proposed mechanism is postsynaptic nitric oxide (NO) mediated signalling. In the visual pathway of tadpoles, it was discovered that endogenous NO synthesis, facilitated by soluble guanylyl cyclase (sGC) initiates cyclic guanosine monophosphate (cGMP) signalling in postsynaptic “V1” neurons. This lead to long term depression (LTD) and associated retrograde spread of LTD to RGCs from the visual processing centre [52]. This retrograde depression observed at RGC synapses was dependant on the activity of protein kinase G (PKG) in RGCs, a molecule known to have degenerative effects in the retina [56]. Given the role reactive oxygen species play in aging, this presents as a strong candidate mechanism for TSD [57]. Another point of note from this study was this retrograde activity depression was also correlated with a decrease in the glutamate receptor subtype, AMPA at RGC synapses, particularly pertinent when considering its prolific role in neurotransmission (Fig. 2).

Lack of afferent input (deafferentation) was explored early on as one possible mechanism for anterograde degeneration [58, 59]. A possible explanation for this is that damage to presynaptic neurons may cause traumatic depolarization and subsequent release of presynaptic glutamate into extracellular interstitium, promoting excitotoxicity within postsynaptic targets [60, 61]. Further, anterograde TSD was still seen without presynaptic trauma by use of genetically manipulated cell loss and thereby loss of presynaptic afferents, leaving open the possibility that it is a lack of afferent input that causes postsynaptic cell degradation. This phenomenon was examined by Triarhou, et al. in Purkinje cells, and by their own admission, the visual pathway was a better model for anterograde transsynaptic atrophy [58]. However, it has been posited that neurotransmission dysregulation rather than outright blockage is responsible [55].

**Figure 3.**
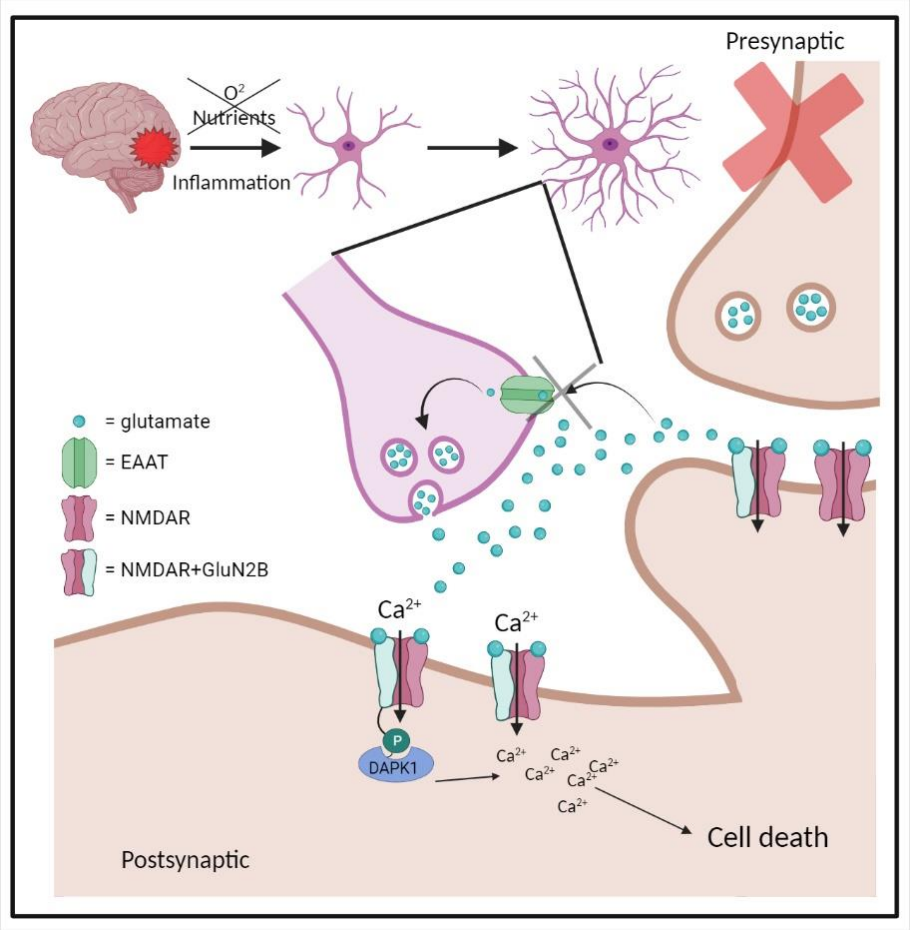
Extrasynaptic NMDAR mediated excitotoxicity by ischemia. Ischemia results in inflammation and a lack of tropic support to cells. This is turn can trigger localised neuronal death, preventing presynaptic glutamate release as well as astrogliosis, leading to reversed glutamate uptake. The resulting glutamate accumulation at the synapse stimulates death-associated protein kinase (DAPK1) activation which drives increased extrasynaptic (but not synaptic) NMDAR conductivity, promoting cell death. excitatory amino acid transporter (EAAT).

Deleglise and colleagues conducted a study to determine whether dysregulated neurotransmission would lead to TSD. To investigate the role glutamate signalling plays in this process, a series of insults to cortico-striatal neuronal networks within a microfluidic environment was employed. The most pertinent being the effect of the application of tetrodotoxin on synaptic activity and whether blockade of cortical activity would trigger spontaneous degeneration of striatal target neurons [55]. This did not result in TSD suggesting that damage is not a result of deafferentation. Despite the lack of change to either cortical axon terminals or striatal dendrites, they do concede that perhaps more chronic blockage (>48 h) is required to see the desired effect. However, while blockade of synaptic transmission alone was not enough to cause TSD, when cortical neurons were bathed in tetrodotoxin prior to insult, the postsynaptic striatal neurons were virtually unharmed by anterograde TSD seen in controls, suggesting transmission is certainly a contributor to damage spread. This is supported by other studies using synaptic blockade to tease out synaptically activated apoptosis mechanisms, with some pathways being dependent on transmission [62, 63]. They posit the mechanism of action for this is that axotomy-driven glutamate excitotoxicity in cortical axons trans-synaptically activates glutamate receptors (NMDARs), facilitating striatal damage and death. This process is specifically mediated by NMDARs containing GluN2B subunit. Similar TSD results were seen in an early study utilising a NMDAR agonist was successfully used as a method of inducing excitotoxic damage from the striatum to the substantia nigra [11]. This is not surprising as glutamate excitotoxicity is a well known contributor to neuronal damage, though it bears noting that NDMARs have been shown to be both protective and damaging [54]. Within the aforementioned study by Deleglise and co, they also investigated ischemic injury to mimic another age-related disease and showed clear evidence of TSD in stroke. This too appeared to be driven by NMDARs, with subsequent inhibition using MK-801 leading to a significant reduction in neuronal pruning. Ischemia specifically increases the extrasynaptic NMDAR activity, triggering downstream death pathways [64]. There is evidence indicating that reverse glutamate uptake by neighbouring astrocytes is a key factor in stimulating this pathway in ischemia, a factor compounded by blockage of presynaptically released glutamate, which did not contribute to downstream toxicity under similar conditions [65, 66]. The extrasynaptic NMDARs containing GluN2B form a complex with death-associated protein kinase 1 (DAPK1) in ischemia and facilitates stroke damage (Fig. 3) [67, 68]. Studies in patients who have sustained stroke damage to the occipital lobe have gone on to exhibit RNFL thinning months to years afterward, suggesting that TSD is bidirectional [8, 69]. This is not the only role synaptic transmission plays in neurodegeneration, disease progression is associated with transsynaptic transmission of toxic proteins along neuronal pathways, a phenomenon frequently observed in AD and PD [70, 71].

#### 3.1.2. Trans-Synaptic Spread of Pathological Molecules

**Figure 4.**
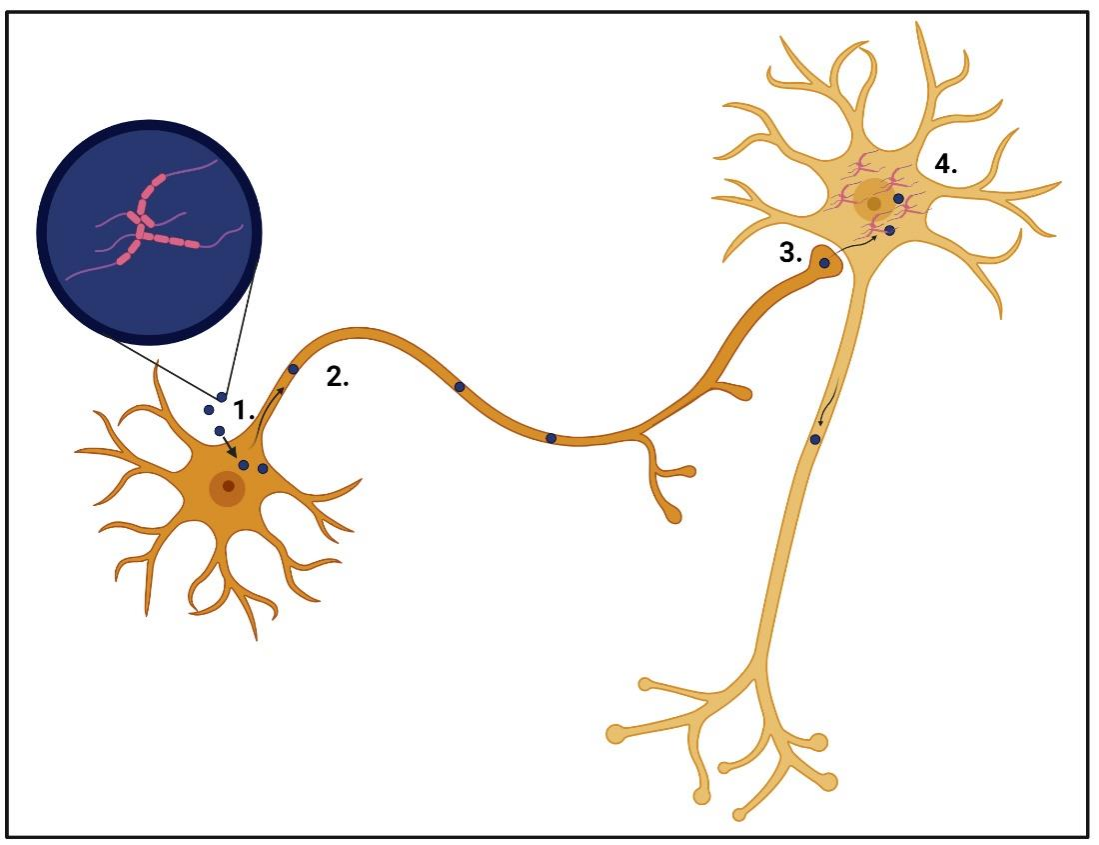
Exosomal transsynaptic transmission of Tau protein in disease conditions. 1. Exosome (blue) containing Tau (pink) are taken up by the cell. 2. Exosomes are transported along the axon to the synaptic terminal. 3. Exosomes are released at the synapse and are taken up by the postsynaptic cell. 4. Tauaggregates begin to form within the cell, leading to eventual cytotoxicity.

Studies have emerged over the last 15 years that provide evidence of AD progression being spread through transsynaptic mechanisms. The deposition of Aβ plaques and Tau neurofibrillary tangles are considered hallmarks of AD, progression is associated with the spread of these throughout the brain via synaptic transmission [70]. A study by Wang and colleagues found that the spread of Tau throughout the brain is achieved by direct transmission of exosomes between neurons [72]. Using cell cultures in a microfluidic chamber, they applied exosomes containing Tau to neurons on the somal side, allowing them to observe the transfer of exosomal Tau to the neuritic side. Their discovery revealed that exosomes containing Tau, released into the extracellular space can be absorbed by neurons. Once internalised, these exosomes undergo axonal transport and are subsequently released at pre-synaptic terminals (Fig. 4). Once released into the synapse, they are taken up by synaptically connected neurons, thereby transporting Tau transsynaptically. For a while, the same was believed to be true for Aβ. While not specifically synaptically exchanged, as Aβ spread seems to be more mediated by extracellular vesicles, there is still a connectivity aspect involved. Further, extracellular vesicles isolated from the CSF of sporadic, late-onset AD patients and animal models had elevated levels of Aβ1-42, not within the vesicle, but located on the external surface, which would be taken up by cortical neurons in culture [73]. Eitan and colleagues found that these neurons, when cultured with Aβ vesicles, were found to destabilise neuronal Ca^2+^ homeostasis and increase susceptibility to excitotoxicity, leading to degeneration and apoptosis.

Another example of TSD through the spread of toxic proteins is seen in Parkinson’s disease (PD). In a mouse model of PD, retrograde transsynaptic spreading of pathological form of α-synuclein (α-Syn) was evident across sensory neurons to pain processing regions [47]. Normally involved in synaptic vesicle recycling, there has been evidence that this misfolded α-Syn begins in the peripheral nervous system (PNS) and is transported to the CNS, behaving in a prion-like manner [74, 75]. In a prior study involving the same PD mouse model, the sciatic nerve was injected with α-Syn fibrils which lead to deposition of it in several brain nuclei connected to motor function [46]. The key finding being that because α-Syn inclusions were present in these nuclei well before they were observed in the spinal cord, is highly indicative of retrograde axonal transport. This is supported and added to by another study showing that α-Syn can operate in a bidirectional transsynaptic fashion. After injection of preformed α-Syn fibrils into the duodenum wall, its depositions were detected in the brainstem and subsequently the stomach, hinting at possible secondary anterograde propagation [76]. Another finding that Ayers and his team encountered in this study was the possibility that the spread of α-Syn pathology may be biased to myelinated axons, likely due to the migration from axons into mature oligodendrocytes during engulfment of diseased axonal segments [77]. This is of significance due to the contribution of glial cells to TSD that will be expanded upon in later sections of this review.

It is also important to note the presence of α-Syn in non-motor PD symptoms, such as vision impairment. α-syn aggregates have been observed in the retina, particularly in ganglion layers where dopaminergic receptors are also expressed [78]. This lends itself to the emerging view that diseases like PD and AD can be diagnosed and tracked through the visual pathway, evidenced by an observed increase in latency of VEPs and retinal thinning in OCT measurements [78, 79]. Similar to PD, there is an accumulation of Tau in the retina and that white matter damage in AD patients extends to the visual system, supporting the notion of vesicular mediated TSD in these diseases [80, 81].

### 3.2. Alterations in Glial Cell Activity

The aging brain develops qualities that make it susceptible to developing neurodegenerative diseases. One of the most prevalent is an increase in inflammatory cells and immune dysregulation, a phenomenon termed “inflammaging” [82, 83]. This wide umbrella covers inflammation, glial activity changes, demyelination, leaky BBB and even senescence is impacted [4]. Accumulating evidence reveals increased immune activity in TSD, providing strong evidence that it is largely an immune-based mechanism driving pathology progression. In TSD conditions, several studies in animal models and humans have demonstrated enhanced glial activity and demyelination that precedes axonal loss [12, 20, 84]. Glial cell response to and release of damage associated molecular patterns (DAMPs) is another important factor to consider when examining the role glial cells play in damage and age-related TSD. DAMPs are released by senescent, stressed, damaged or dying cells, and are detected by immune receptors on glial cells [85]. Therefore, it is crucial to explore the impact of inflammaging on glial changes to comprehend the underlying degenerative mechanisms of age-related neurodegenerative disease.

#### 3.2.1. Astrocytes

**Figure 5.**
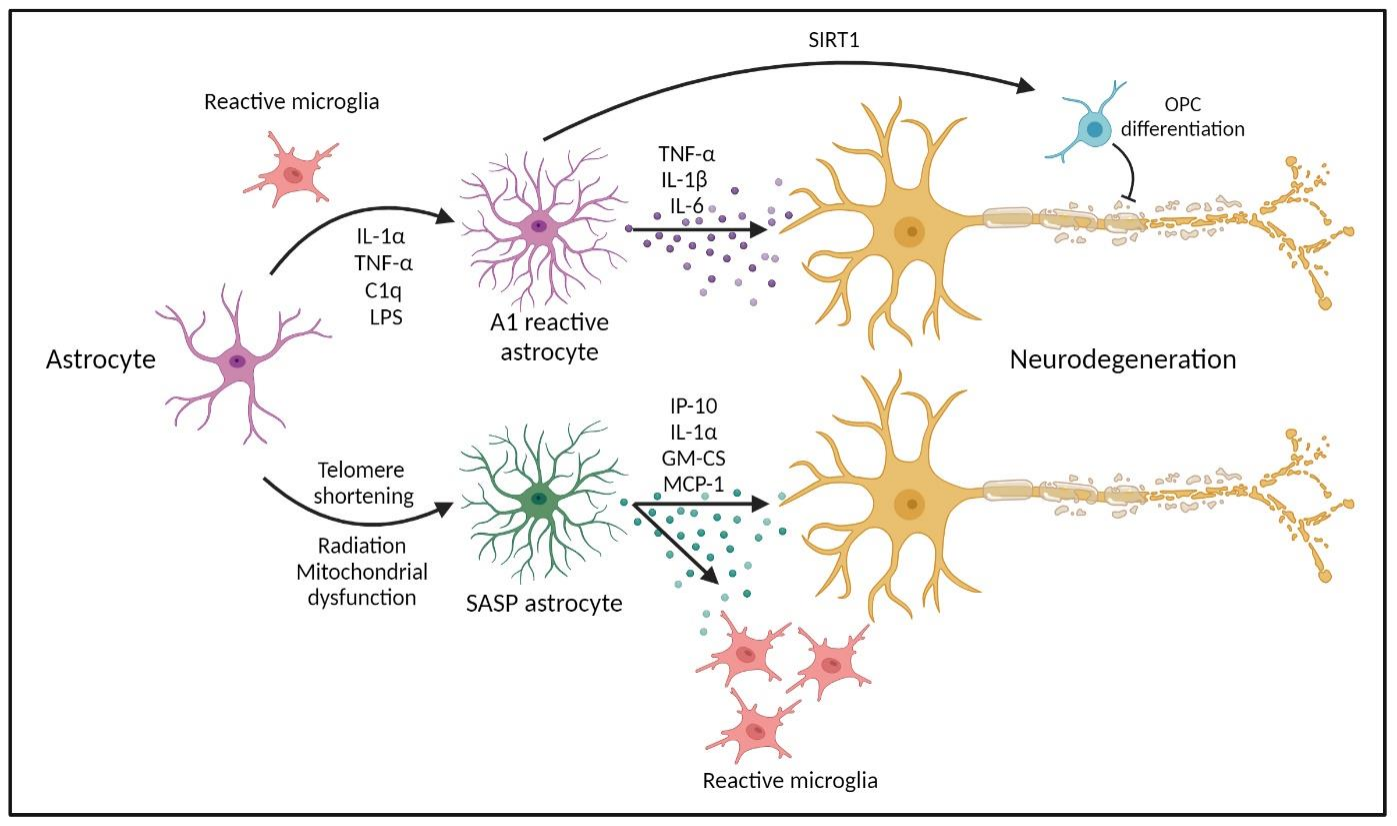
Reactive vs Senescent Astrocyte activity. Pro-inflammatory mediators (IL-1a, TNF-a, and C1q or LPS) released by activated microglia generate reactive astrocytes. Reactive astrocytes express C3 and secrete pro-inflammatory cytokines (TNF-a, IL-1b, and IL-6), while senescent astrocytes secrete the SASP (IP-10, IL-1a, GM-CSF, and MCP-1), activating microglia, both of which are related to the progression of neurodegenerative diseases.

When a living organism ages, cells in the tissues undergo senescence, and this process occurs when the telomeres are worn down, resulting in loss of cellular proliferative capacity [86]. Usually a beneficial process, this has also been seen to contribute to age-related pathologies such as senescence-associated secretory phenotype (SASP) and secretion of DAMPs, particularly high mobility group box 1 protein (HMGB1). Under these conditions, the glial phenotype exhibits increased expression and secretion of proinflammatory cytokines and chemokines, causing changes in the CNS immune landscape (Fig. 5) [7, 87, 88]. This is of particular interest with regard to astrocytes, as they are the dominant glial cell type in the CNS [89]. Under normal conditions, astrocytes play a major role in CNS homeostasis, providing neuronal support, BBB regulation, control of neurotransmitter clearing and recycling, synapse modulation and immune defence [90]. However, its role as a neuroprotector begins to change with age. During neuroinflammation, reactive astrogliosis occurs, converting them to neurotoxic type A1 astrocytes, decreasing the excitatory function of neurons and formation of new synapses, potentially leading to cell death [91]. Though A1 phenotype is different to the SASP, it bears a different secretory profile to A1 and are activated by stressors such as DNA damage and oxidative stress and are capable of triggering neuroinflammation. This then contributes to HMGB1 levels and its extracellular release, resulting in upregulation pro-inflammatory cytokines and priming of astrocytes [92].

An experiment conducted by Kawano and colleagues demonstrated that healthy neurons, when co-cultured with aged astrocytes, exhibited altered synaptic function [93]. This affected the presynaptic neurons such that the readily releasable pool of vesicles was diminished, with no subsequent activity seen in postsynaptic neurons. This contributes to the idea that anterograde TSD could be, at least partially, synaptically mediated. While Kawano’s team did not note any neuronal damage, this is different to a study by Limbad and colleagues, which documented excitotoxic neuronal death [94]. Because the senescent astrocytes cannot adequately manage the excess extracellular glutamate, this leads to cell death. Both A1 and senescent astrocytes exhibit a downregulation in their excitatory amino acid transporters (EAAT), contributing to their possible role in excitotoxic TSD (Fig. 3) [95]. However, rather than allow the astrocytes to age and reach replicative senescence naturally, Limbad induced it using X-irradiation. This presents the question of whether Kawano’s cells were truly senescent or merely A1 astrocytes. Seeing as they did not test for SASP markers like Limbad did, it is possible Kawano and colleagues were looking at A1 reactive astrocytes, though these still play a part in age-related TSD [91, 96, 97].

Increased markers of astrocyte senescence have been identified in AD. Characterized by extracellular deposition of beta amyloid (Aβ) peptide and intra-neuronal accumulation of phosphorylated Tau, in vitro studies indicate that these two peptides can cause a healthy astrocyte to become senescent., Further, AD sufferers showing greater senescence than age-matched counterparts [90, 98]. A similar trend is seen in PD, with neurotoxic protein α-Syn induced senescence in both astrocytes and microglia [99]. Using a combination of α-Syn preformed fibrils (PFF) treated cells, mouse models and human PD patient brains, it was discovered that α-syn PFF-induced cellular senescence led to toxic protein-aggregation based neuronal loss. In addition to SASP, astrocytes acquired A1 reactive phenotype, triggered by microglial secretions of interleukin 1α (Il-1α), tumour necrosis factor (TNF), and complement component 1, subcomponent q (C1q) in animal models [97, 100]. A recent study showed that blocking of microglial-mediated astrocyte conversion with glucagon-like peptide-1 receptors (GLP1R) agonist, NLY01, exhibited protective effects in this PD model and successfully protected against dopaminergic neuronal loss and behavioural deficits [101].

Senescent astrocytes also have a strong impact in MS conditions by playing an active role in de/remyelination processes. Senescent astrocytes prevent differentiation of oligodendrocyte precursor cells (OPCs), thereby hampering tissue repair and this is largely mediated through the protein, sirtuin 1 (SIRT1) [102-104]. One of the functions of astrocytes in demyelination conditions is to clear the damaged oligodendrocytes or myelin layer to make room for OPCs to move in and replace them. However, in disease conditions like MS, astroglial scarring in addition to preventing access of cytokines and other inflammatory agents, also inhibits neurotrophic and repair factors from reaching sites of injury, impairing the remyelination process [105, 106].

#### 3.2.2. Microglia

The senescence state is also observed with microglia. Healthy microglia are the first line of defence in the CNS, responding to damage or infection, but also play a vital role in tissue homeostasis [107]. In response to primary insult DAMPs are released by damaged or dying cells, which are detected by immune receptors of microglia. The resulting localised neuroinflammatory response can damage surrounding neurons, contributing to TSD [108]. While microglia do exhibit the key telomere shortening and corresponding DNA damage indicative of senescence, they do not share the same SASP profile as astrocytes. Some studies have also reported immune activation of microglia by HMGB1 through signalling pathways [92]. With the populations of activated and senescent microglia overlapping, it is difficult to differentiate the two phenotypes, as it is unclear whether senescence upregulates markers associated with activated microglia or downregulates the homeostatic markers [109-111]. What is known however, is that in aging and neurodegenerative diseases, they are mainly characterised by a proinflammatory phenotype and a loss of homeostatic function [112]. Normal physiological microglial activity is changed in several neurodegenerative disease conditions. Microglial dystrophy precedes the spread of Tau pathology, with senescent microglia reliably colocalised with Tau neurofibrillary tangles in AD patients [113]. In the Braak rat model, Streit and colleagues demonstrated microglial mediated TSD was associated with the anatomical spread of Tau, showing the presence of senescent microglia in regions of neurodegeneration before the onset of neurofibrillary pathology.

TSD plays a critical role in diseases like MS, leading to disease progression and clinical deterioration [30, 114]. Though not necessarily considered an aging-related disease, MS can still be examined to help tease out important details about the mechanisms of TSD. TSD is often observed in the visual pathway in MS patients, frequently as the presenting symptom [20, 30, 115]. Given that MS is as an autoimmune disease, the first and most prolific mechanism of action is inflammation. Demyelination leaves axons open to attack by glial cells and inflammatory factors, secondary ischemic injury and lack of trophic support [116]. As previously mentioned, microglia, behaving like sentinels to monitor the environment and respond accordingly In the event of infection or neuronal damage, microglia will migrate to the site and ready an immune response or begin clearing oligodendrocyte debris by phagocytosis respectively [32]. This is extremely important for allowing remyelination to occur as it clears the site for OPCs to gain access to begin repair and secrete growth factors [117, 118]. However, dysregulation of their normal activity and acquisition of senescent phenotype might contribute to TSD in MS pathology [119].

In an animal model of MS, abnormal activity of microglia was observed long before gross demyelination of axons and even continued to grow in number until demyelination was complete [120]. This implies that they may be phagocytosing or mounting an immune response against healthy myelin sheaths involved in TSD process. This study was informative not only for what it revealed about the activity of microglia under demyelinating conditions, but also because it removed confounding interference from leukocytes infiltrating the blood brain barrier.

#### 3.2.3. Oligodendrocytes

**Figure 6.**
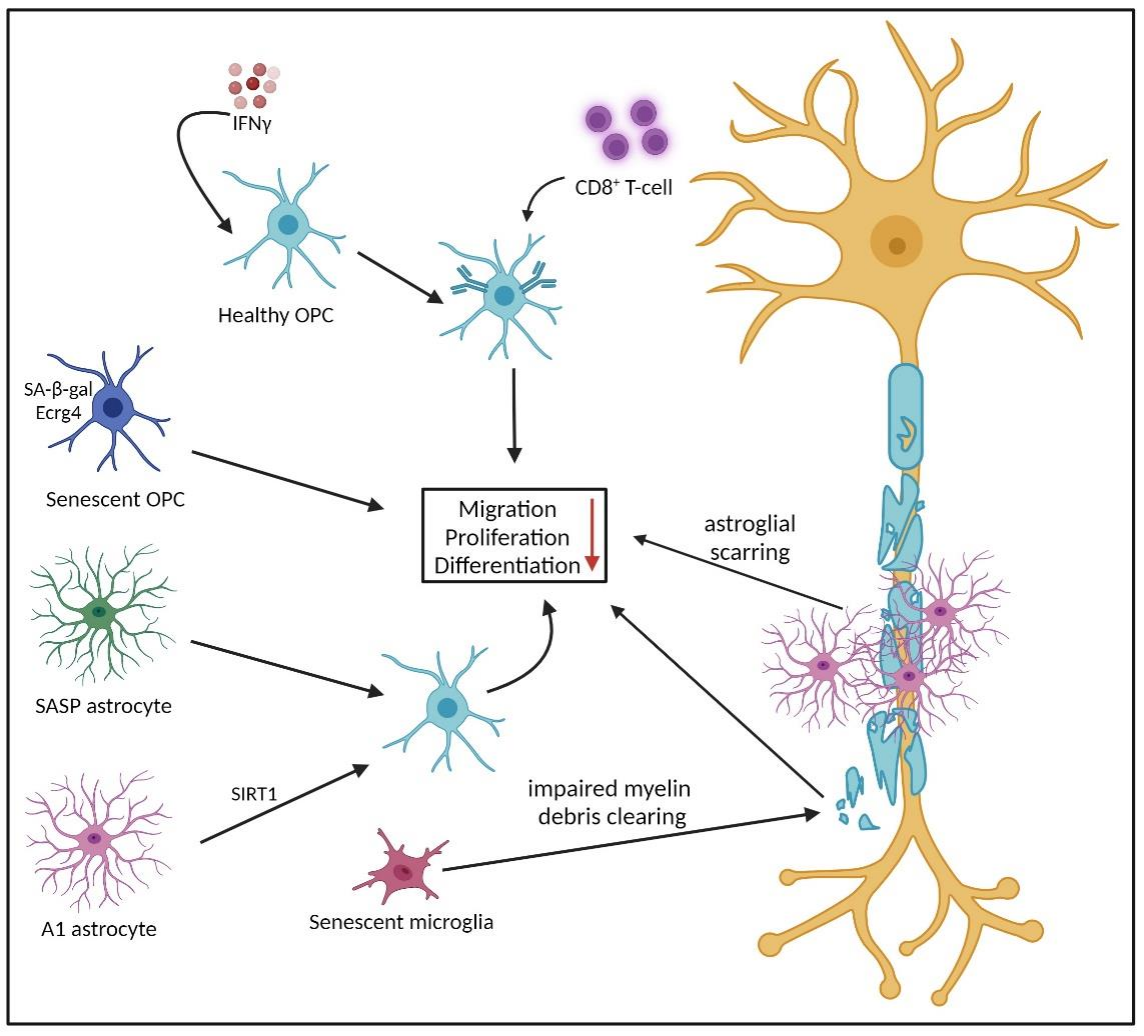
Remyelination impairment in the aging brain. IFNγ promotes antigen cross-presentation in OPCs, this activates cytotoxic CD8+ T-cells, which then target OPCs. A1 astrocytes and senescent glial cells of all types impact remyelination These ultimately prevent proliferation, differentiation and migration of OPCs, thereby preventing remyelination, eventually leading to neuronal death.

Oligodendrocytes are the ultimate axonal support glia, boasting insulation, saltatory conduction, intra-axonal transport and trophic support [121]. Malfunction of these cells exerts a profound effect on TSD. In aging, there is a general decrease in white matter, and therefore volume, in the nervous system, thought in part to be mediated by CD8^+^ T-cells [122]. Post mortem sampling of white matter lesions and normal white matter showed that glial dysfunction is not restricted to lesions, implicating TSD [123]. This is unsurprising as a single oligodendrocyte can form up to 40 myelin internodes around different axons [124]. If inflammatory gliosis contributes to oligodendrocyte death, this could affect a large number of neurons and contribute to TSD process. Senescence-associated β-galactosidase (SA-β-gal), a marker for oxidative DNA damage senescence is observed in oligodendrocytes, suggesting that they are subject to stress-associated cellular senescence in aging individuals [20, 30, 115]. Upregulation of SA-β-gal has also been seen in senescent OPCs, along with oesophageal cancer-related gene 4 (Ecrg4). It has been shown in a rodent model that overexpression of Ecrg4 in OPCs induced senescence via acceleration of proteasome-dependent degradation of cyclins D1 and D3 (Fig. 6) [125, 126]. It is thought that OPCs are solely responsible for remyelination given that, even mature oligodendrocytes that survive demyelination, do not contribute to the remyelination process [127]. In addition to SIRT1, HMGB1 also plays a role in preventing OPC differentiation. A recent study demonstrated that release of HMGB1 by senescent progenitor cells from multiple sclerosis patients impaired maturation of OPCs to myelinating oligodendrocytes [128]. This contributes to the theory that senescent cells help drive TSD.

There has even been evidence that OPCs can be co-opted into perpetuating the autoimmune response in lesions of MS brains, preventing remyelination. Kirby and team identified a signalling pathway in OPCs in vitro that induces antigen-presenting capability through induction of the immunoproteasome as well as prevents them from maturing and subsequently remyelinating. Similar to oligodendrocytes, OPCs are sensitive to IFNγ, which promotes antigen cross-presentation in OPCs, activating cytotoxic CD8+ T-cells, which then turn on OPCs. Similar phenotypic changes were observed in post mortem MS white matter lesion tissue [129].

It has also been shown that demyelination is a precursor to axonal loss [12]. In a longitudinal study of MS patients with unilateral optic neuritis (ON), You and team measured the effect of chronic optic nerve demyelination in patients with progressive RGC axonal loss. They assessed the demyelination in the optic nerve using non-invasive VEPs and retinal thinning using OCT. They found that while both ON and non-ON eyes exhibited rapid RGC loss compared to healthy individuals, the RNFL of ON eyes showed accelerated thinning compared with non-ON fellow eyes, providing strong evidence of TSD.

Glaucoma is another age related retinal degenerative disease condition resulting in loss of RGCs, optic nerve head damage and ultimately leads to blindness in older individuals. In experimental models of glaucoma, anterograde TSD is seen in the LGN and visual cortex of the brain, characterised by glial activation and apoptotic markers [9, 130]. Dysregulated glial activity has been shown in the higher visual centres after optic nerve damage conditions in several studies [31, 131, 132]. This is of particular significance as this implies the spread of degeneration over second and third order of neurons in the visual pathway, passing through two synaptic connections.

Another interesting feature of glaucoma is that RGC apoptosis is thought to be resultant of a decrease in trophic support from their target neurons in the posterior visual pathway following optic nerve injury [133]. Nerve growth factors, promote survival and differentiation, is provided by retrograde/anterograde transport along optic nerve, their blockage making it a possible contributor to synaptically mediated TSD [134, 135].

**Figure 7.**
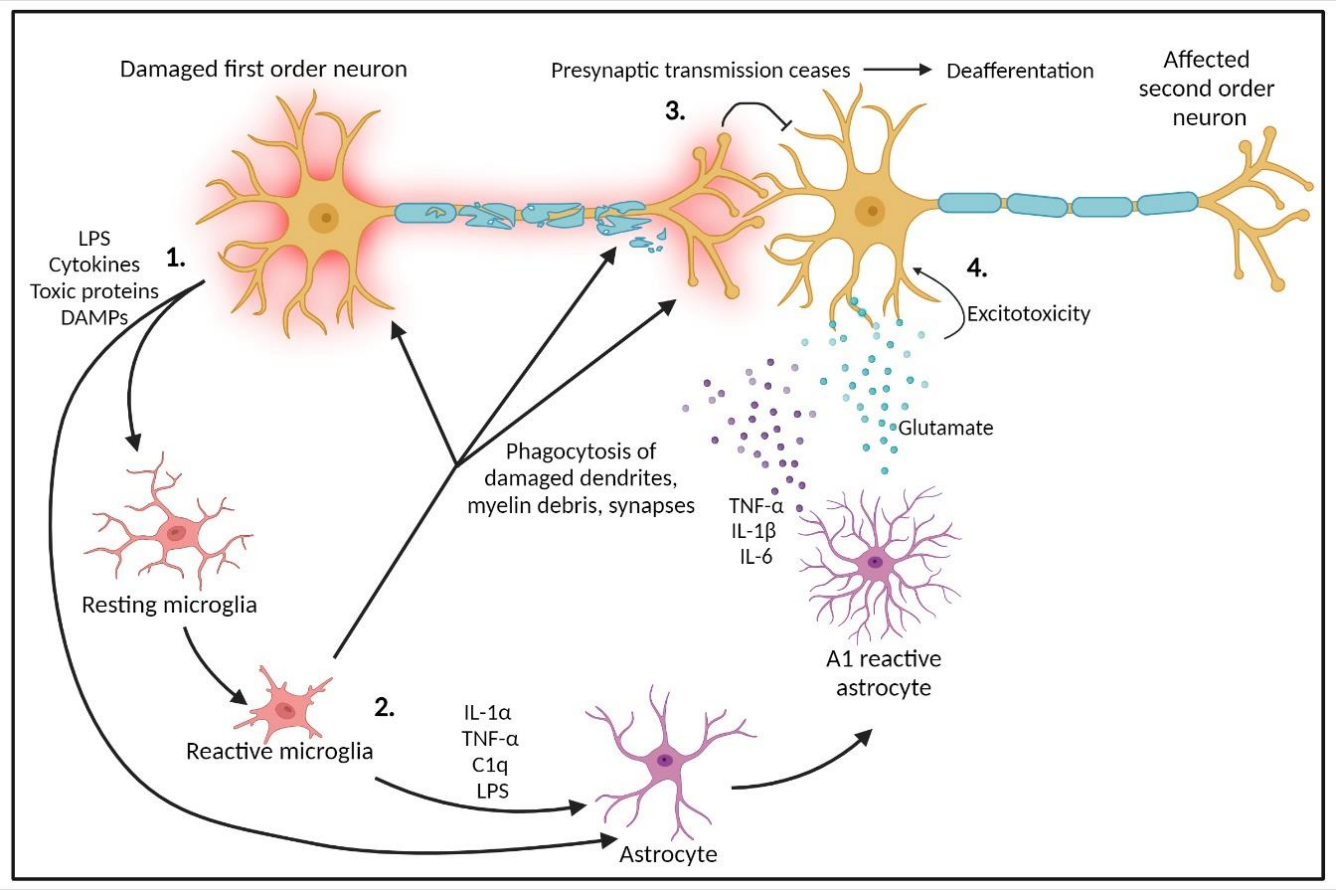
The combined role of glial cells in TSD. Rather than one mechanism in glial reactivity being responsible, it is more likely that it is a combinatorial effect, incorporating deafferentation and excitotoxicity. 1. Damaged first order neurons secrete cytokines, DAMPs and toxic proteins such as α-Syn, which then activate microglia. 2. Reactive microglia then phagocytose dendrites, myelin debris and synapses of damaged first order neurons. They also convert astrocytes to the A1 phenotype. 3. Damaged or phagocytosed synapses of the first order neuron cannot transmit to the second order neuron, contributing to deafferentation. 4. Lack of presynaptic transmission is overcompensated for by the excessive glutamate release from A1 astrocytes, contributing to postsynaptic excitotoxicity. A1 astrocytes also release inflammatory cytokines that can damage the second order neuron.

Given the strong presence of aberrant glial activity in these neurodegenerative diseases, in which TSD is commonly overserved, it is not difficult to draw the conclusion that TSD is largely a glially mediated process. However, there are data that this may not be the sole mechanism, with many of the aforementioned studies championing the theory that it could be a synaptically driven process (Fig. 7).

## 4. A Favoured Direction for Each Mechanism?

From the studies mentioned in this article, it would appear that some mechanisms of TSD spread damage in a preferred direction, though not always exclusively. Neurotransmission and excitotoxicity exhibit different mechanisms that can spark degeneration in either direction. Glutamate excitotoxicity can cause anterograde degeneration though hyperstimulation of postsynaptic neurons, but it is also capable of causing retrograde degeneration by triggering back propagation [11, 53, 55]. Similarly, the direction of vesicular spread of pathological proteins appears to differ depending on the disease. For instance, Aβ exosomal transfer tends to occur in the anterograde direction, and though there are some mentions of it moving anterogradely, α-Syn predominantly moves in a retrograde manner [46, 72]. Similarly, dysregulated glial mediated TSD is also challenging to pin to a direction. Conforming with the Braak spread of AD pathology, it appears that microglial mediated TSD operates in an anterograde direction, preceding Tau spread [113]. Similarly in glaucoma, upregulated glial activity appears to correlate strongly with anterograde TSD as well [9, 31, 47]. Though visual pathway disorders such as ON and stroke or damage in the V1 show a strong prevalence of retrograde TSD, there is still plenty of evidence of anterograde spread after these instances [27, 31, 55, 136-138]. Being able to determine patterns of TSD spread in neurodegenerative diseases could help mitigate the progression to other areas, though this is quite challenging as several major mechanisms appear to move bidirectionally depending on the situation.

## 5. Potential therapies

The most prominent caveat surrounding neurodegenerative disease research is determination of the initial trigger. It has been unclear for decades whether it is propagated spontaneously or by some insult, leading to an ongoing, cyclical response. There are a multitude of potential strategies that may develop into treatments with the aim to slow, halt or even reverse TSD progression.

### 5.1. Neurotransmission and Synaptic Targeting

#### 5.1.1. Excitotoxicity

As GluN2B-NMDARs driven excitotoxicity is a prevalent feature among multiple neurodegenerative diseases, including AD and PD, the use of NMDAR antagonists could act in a neuroprotective manner [55]. As NMDAR associated toxicity has been narrowed down to the Glun2B subunit, several studies on the design and application of GluN2B antagonists have been conducted [54, 139]. Much progress has been made with the specificity of these antagonists, leading to enhanced understanding of GluN2B-containing NMDAR function and its related complexes. There are four prominent signalling complexes that are being explored, each of which is responsible for mediating neuronal death and/or LTD. Mentioned previously, GluN2B- DAPK1 is a neuronal death signalling complex which, when deleted or uncoupled from the NMDAR subunit, is protective against ischemic injury [67, 140]. This makes it a promising therapeutic target for mitigating TSD in stroke damage.

The combination of Ras protein specific guanine nucleotide Releasing factor 1 (RasGRF1) with GluN2B-containing NMDARs allows it to activate the p38 mitogen-activated protein kinase (MAPK) signalling pathway, which plays critical roles in LTD and excitotoxicity [68]. Though this protein has neuroprotective functions as well, its possible role in neuronal death signalling requires further investigation. Inhibition of another protein that forms a complex with GluN2B, CaMKII, with similar properties of both protective and destructive functions, has recently been found to be neuroprotective against glutamate-induced excitotoxicity when applied before or shortly after injury [141].

The GluN2B-postsynaptic density protein 95 (PSD95)-neuronal nitric oxide synthase (nNOS) complex signalling pathway mediates neuronal death. A number of drug studies have been conducted to interrupt the formation of this protein complex during neuronal insult, with these therapeutics providing neuroprotection in rodent models of ischemic stroke. There is even some evidence that it may contribute to synaptic plasticity, which may provide other avenues of treatment development [142]. Despite the effective research being conducted on these receptors and complexes in animal models, translation to a clinical setting has been largely unsuccessful [143]. This stalemate has lead to the development of positive allosteric modulators (PAMs) in an attempt to manipulate synaptic plasticity in neurodegenerative diseases, with some ongoing trials in PD (NCT05318937) and AD (NCT05619692) to improve cognitive symptoms [68].

#### 5.1.2. Vesicle Mediated Protection

Extracellular vesicles themselves are becoming the primary focus in therapeutic strategies for vesicular-mediated diseases such as AD and PD. It is possible to utilise the extracellular vesicle proteins as biomarkers of disease [71]. It was evident that extracellular vesicle-bound Aβ collected from AD patient plasma was more closely linked to brain plaque deposition (assessed by positron emission tomography scan) than amount of total plasma Aβ, demonstrating its use as a peripherally detectable biomarker [144].

Ability of exosomes to sequester Aβ in vivo to rescue synaptic function and plasticity, was demonstrated in a study. The exosomes from either N2a cells or healthy human cerebrospinal fluid, were injected into rodents along with a subsequent injection of either synthetic or human AD brain derived Aβ [145]. It has been observed that, rather than Aβ proteolysis as the clearing mechanism, synaptotoxic Aβ assemblies were sequestered by exosomal surface proteins such as cellular prion protein and glycosphingolipids [146].

Reducing or blocking the transsynaptic spread of toxic α-Syn is a method currently being explored as a means of slowing PD progression. An anti-α-Syn target, proSAAS, has been found to block α-Syn-induced dopaminergic cytotoxicity in rodent models [147]. The small protein, proSAAS, is a neuronal chaperone stored in secretory vesicles, specifically secreted to eliminate pathogenic aggregates, shown to block the aggregation of both α-Syn and Aβ1-42 in vitro [148, 149]. It is possible that it not only mitigates toxic aggregate accumulation but may have the ability to maintain normal α-Syn vesicle cycling machinery. Further understanding into its exact mechanism offers a potential promising therapeutic target to prevent the spread of TSD.

### 5.2. Targeting Glial Cells

#### 5.2.1. Senescence

Modulation of the overwhelming reactivity of glial component in TSD offers a promising treatment strategy to prevent the spread of damage. GLP1R is expressed at high levels in both microglia and astrocytes and its modulation exhibited neuroprotective effects in neurodegenerative disease conditions [97, 150]. An enhanced colocalization of GLP1R was seen with microglial marker, IBA1 in the substantia of α-Syn PFF animals [101]. Neuroprotective effects were observed when GLP1R agonist supressed the microglial induced activation of astrocytes to the A1 toxic phenotype in this mouse model. Similar effects were found when microglial activity was suppressed with monoamine oxidase inhibitor in an AD mouse model [151]. Tranylcypromine, a commercially available drug was used to modulate Aβ-induced microglial activation, though the exact mechanism currently remains unknown. Though, to truly address the age-related aspect, senescence targeting could be highly beneficial [87]. A recent study in older adults has shown that circulating SASP biomarkers were reduced with exercise [152]. This is supported by senescent microglial changes in aged mice, showing a reduction in senescent markers in elderly mice that received more exercise compared to more sedentary controls. Mela and colleagues were able to demonstrate that neuroinflammation and poor metabolic performance in microglia was attenuated with a steady exercise regime [153]. Though the exact mechanism through which exercise is achieving this process is yet unknown, it is a promising avenue.

#### 5.2.2. Remyelination

Remyelination stimulation is a popular strategy currently being researched with some extent of success in MS models [84, 154]. To combat remyelination dysfunction in an aged animal model, the use of Metformin was able to reverse age-related changes in OPCs, prompting their responsiveness to differentiation factors thus rejuvenating poor remyelination in aged rodents [155]. In this study, it appeared that metformin, a commercially available fasting mimetic was able to restore OPC differentiation via the AMPK pathway, a central nutrient signalling pathway that also aides in mitochondrial function. For pathologies impacting the visual pathway, it is now far easier to monitor disease progression through the use of VEPs as an end point in clinical trials as it is far less invasive. The use of VEPs to assess metformin action in focal demyelination showed successful conductivity protection in the visual pathway compared to controls [156]. This drug also has an positive effect on mitigating activated microglia in demyelinating models, and given its status as an FDA approved substance, this could prove an extremely effective therapeutic for neuroinflammatory diseases [157].

Another possible remyelination target has been found in a recent study illustrating the neuroprotective role of SIRT1 blockade in a chronic demyelination model. SIRT1 expression by reactive astrocytes has been shown to prevent OPC differentiation, though its inactivation resulted in inhibited the production of proinflammatory mediators by microglia and promoted differentiation of OPCs [102, 158]. This has prompted calls for its use as a possible therapeutic target for inflammatory neurodegenerative diseases such as MS, PD and AD [159].

Many of these neuroprotective methods can be combined and applied across neurodegenerative diseases as they share a good portion of the destructive mechanisms. Many revolve around trying to maintain the normal, supportive role of glial cells and block their more damaging phenotype from emerging or acting. Though many of these therapies are still in their infancy and more development is needed for human application, it is encouraging that advances are being made in understanding the mechanisms of action of this multifaceted phenomenon.

## 6. Conclusions

Transsynaptic degeneration is a well-documented phenomenon across various neurodegenerative disease conditions. A large number of age-related neurodegenerative diseases appear to share core mechanisms of development and progression. The immune dysregulation accounts for a significant portion of it and gives all the more credence to the glial-mediated theory of TSD. However, synaptic activity driven spread of damage cannot be ignored. More than likely, it is a combination of the two, though more wholistic approaches to this problem are required. Studies combining the glial aspects and the synaptic activity in various models of TSD related disease could provide much needed deeper insight into progression and potential treatments.
